# Successful expression of the *Bordetella petrii* nitrile hydratase activator P14K and the unnecessary role of Ser115

**DOI:** 10.1186/s12896-016-0252-2

**Published:** 2016-02-20

**Authors:** Weifeng Sun, Longbao Zhu, Xianggui Chen, Ping Chen, Lingling Yang, Wenwu Ding, Zhemin Zhou, Yi Liu

**Affiliations:** Key Laboratory of Food and Biotechnology, School of Food and Biotechnology, Xihua University, Chengdu, 610039 China; School of Biochemical Engineering, Anhui Polytechnic University, Anhui, 241000 China; Key Laboratory of Industrial Biotechnology, School of Biotechnology, Jiangnan University, Wuxi, 214122 China

**Keywords:** NHase, *Bordetella petrii*, P14K, Expression, Ser115

## Abstract

**Background:**

The activator P14K is necessary for the activation of nitrile hydratase (NHase). However, it is hard to be expressed heterogeneously. Although an N-terminal strep tagged P14K could be successfully expressed from *Pseudomonas putida*, various strategies for the over-expression of P14K are needed to facilitate further application of NHase.

**Results:**

P14K was successfully expressed through fusing a his tag (his-P14K), and was over-expressed through fusing a gst tag (gst-P14K) at its N-terminus in the NHase of *Bordetella petrii* DSM 12804. The stability of gst-P14K was demonstrated to be higher than that of the his-P14K. In addition, the Ser115 in the characteristic motif CXLC-Ser115-C of the active center of NHase was found to be unnecessary for NHase maturation.

**Conclusions:**

Our results are not only useful for the NHase activator expression and the understanding of the role of Ser115 during NHase activation, but also helpful for other proteins with difficulty in heterologous expression.

## Background

Nitrile hydratase (NHase) is a multi-subunits industrial enzyme, which can efficiently catalyze the nitrile to the corresponding amide. According to the different metal ions in the active center, the NHase could be classified into the two types: the iron type NHase (Fe-NHase) [1] and the cobalt type NHase (Co-NHase) [2]. Due to the high-value product and high catalytic activity, NHase has attracted wide attention in the chemical industry [3].

NHase is composed of α- and β-subunits [2]. The molecular masses of the α-subunit and β-subunit are generally less than 30 kDa, such as those in *Bordetella petrii* DSM 12804 (α-subunit, 23.2 kDa; β-subunit, 24.1 kDa) and those in *Bacillus pallidus* RAPc8 (α-subunit, 24.6 kDa; β-subunit, 26.5 kDa) [4, 5]. However, an activator is necessary for the functional expression of NHase, such as those in the Co-NHases from *Rhodococcus rhodochrous* J1 [6–8], *Rhodococcus jostii* RHA1 [9] and *Pseudomonas putida* NRRL-18668 [10] as well as in the Fe-NHases from *Pseudomonas chlororaphis* B23 [11] and *Rhodococcus sp*. N-774 [12]. However, certain activators are poorly expressed (barely detectable in SDS-PAGE), such as the activator P14K in the *B. pallidus* RAPc8, *Comamonas testosteroni* 5-MGAM-4D and *P. putida* NRRL-18668 [4, 10, 13]. Until recently, the P14K in *P. putida* was successfully expressed through fusing a strep tag at its N-terminal, then a novel NHase maturation mechanism, self-subunit swapping, was discovered [5]. However, the stability of the P14K is not yet favorable for its over-expression. Thus, to further facilitate the application of NHase, other fusion protein tags that are helpful for expression of more stable P14K are required.

**Fig. 1:**
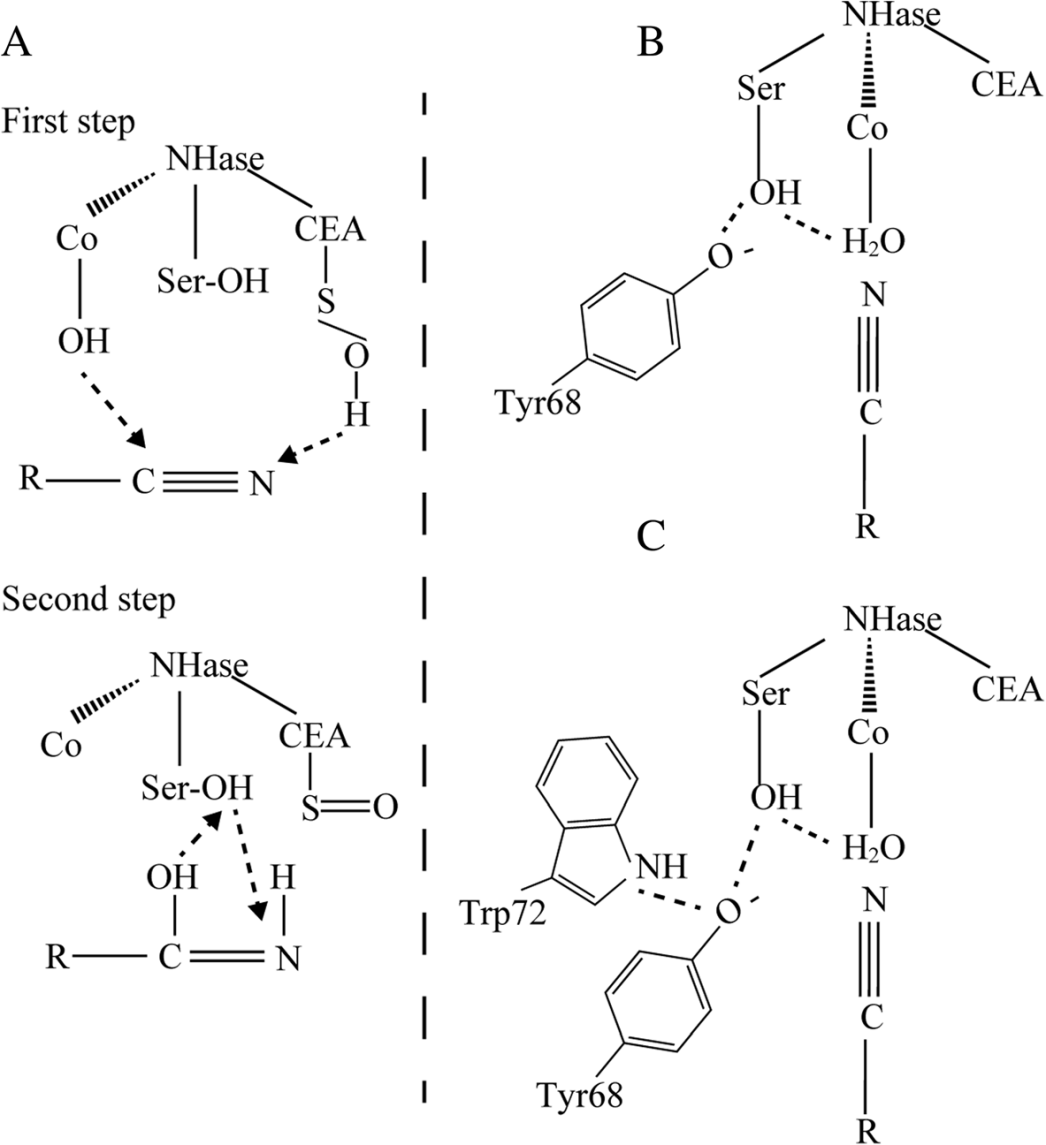
The catalytic pathway involving Ser115 during NHase maturation. **a** First step: atoms of O(CEA)-H(H_2_O) and OH(H_2_O)-Co^3+^ participates in NHase activation (*dashed line*). Second step: the proton of OH(Ser115) participates in the proton transfer pathway (*dashed line*). **b** The oxygen of the phenolate of the conserved Tyr68 was ionized by the general base Ser115 during NHase activation. **c** The oxygen of the phenolate of the conserved Tyr68 was ionized by the general base Ser115 with the help of Tyr72 during NHase activation

Additionally, all the α-subunits of NHases share a conserved characteristic motif CXLC-Ser-C in the active center [1, 14, 15], and the Ser in this motif is proposed to be involved in NHase maturation (Fig. 1) [16–18]. However, the necessity of Ser was not studied by the energetic calculation and site-specific mutagenesis.

In this study, the NHase from *B. petrii* DSM 12804 was researched. The activator P14K was successfully expressed through fusing a his tag, and its expression and stability were further enhanced by fusing a gst tag at its N-terminus. In addition, we found that the Ser115 in the characteristic motif CXLC-Ser-C of the active center of NHase from *B. petrii* is not necessary for the NHase maturation*.* These results are useful for the expression of NHase activator and the understanding of the role of Ser115 during the NHase maturation. Further, the strategy for enhancing the stability of P14K is very beneficial for other proteins with difficulty in expression.

## Results and discussion

### Expression and purification of NHase

**Fig. 2:**
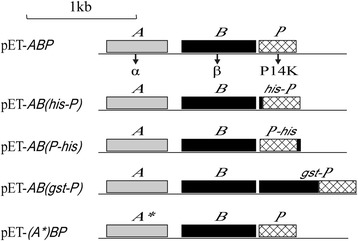
Sketch of plasmids construction. pET-*ABP*, a plasmid including NHase encoding genes *A* (α-subunit), *B* (β-subunit) and *P* (P14K); pET-*AB(his-P)*, a plasmid including NHase encoding genes *A* (α-subunit), *B* (β-subunit) and *his-P* (N-terminal his-tagged P14K); pET-*AB(P-his)*, a plasmid including NHase encoding genes *A* (α-subunit), *B* (β-subunit) and *P-his* (C-terminal his-tagged P14K); pET-*AB(gst-P)*, a plasmid including NHase encoding genes *A* (α-subunit), *B* (β-subunit) and *gst-P* (N-terminal gst-tagged P14K); pET-(A*)BP, a plasmid including NHase encoding genes A* (mutant α-subunit with Ser115 to Ala115), *B* (β-subunit) and *P* (P14K)

**Fig. 3:**
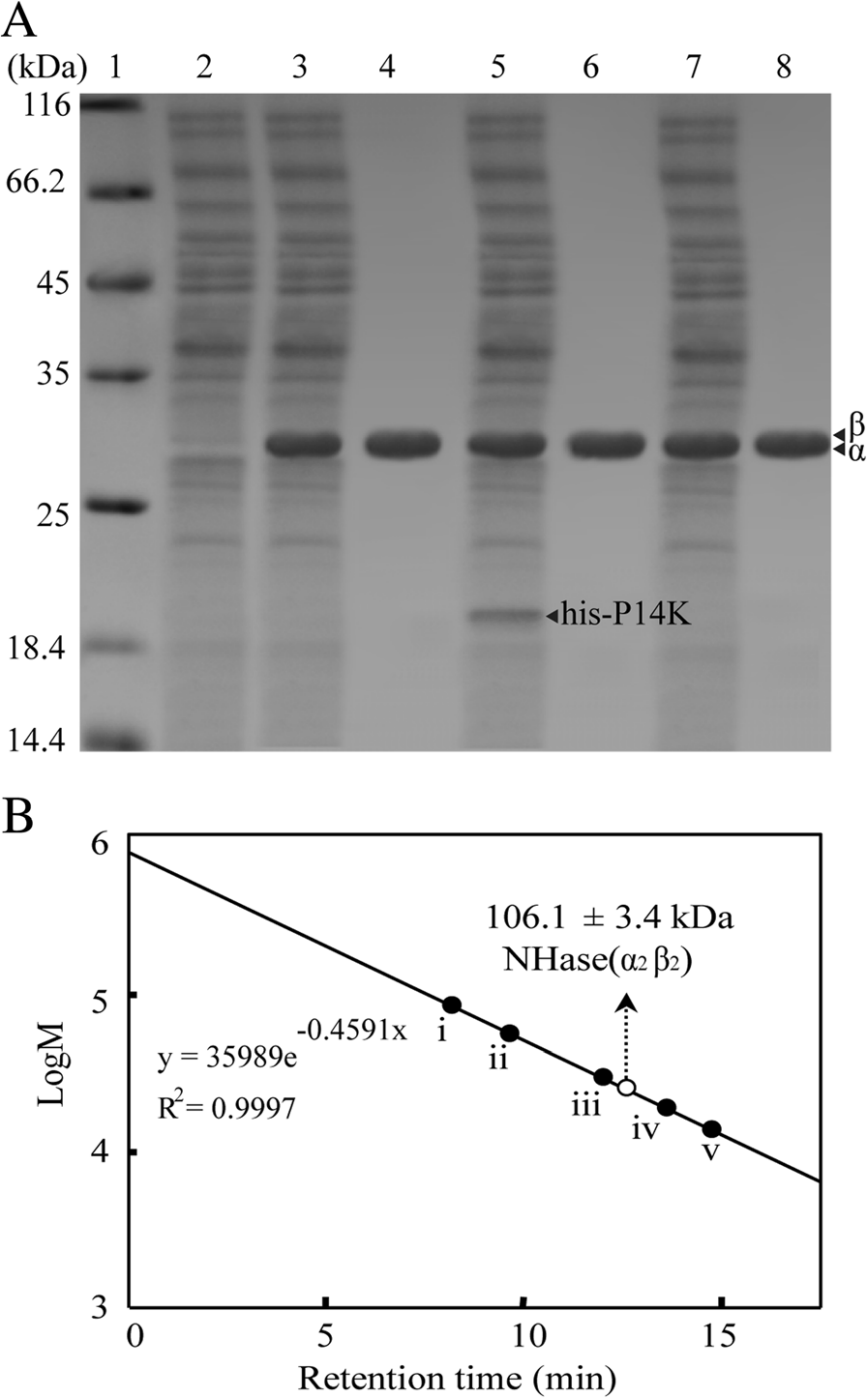
Expression and purification of recombinant NHases. **a** 1, mark; 2, control; 3, cell extracts (pET-*ABP*); 4, purified NHase (pET-*ABP*); 5, cell extracts (pET-*AB(his-P)*); 6, purified NHase (pET-*AB(his-P)*); 7, cell extracts (pET-*AB(P-his)*); 8, purified NHase (pET-*AB(P-his)*). **b** Molecular mass determination on a Superdex 200 prep grade column. Standard proteins are as follows: (i) thyroglobulin (bovine thyroid) (669 kDa); (ii) ferritin (horse spleen) (440 kDa); (iii) aldolase (rabbit muscle) (158 kDa); (iv) conalbumin (chicken egg white) (75 kDa); and (v) ovalbumin (hen egg) (43 kDa). The open circle represents the determined molecular mass. The values represent the means ± SD for at least three independent experiments [5]

The full-length genes of NHase (*ABP*) were obtained from *B. petrii* DSM 12804, in which *A* gene encodes α-subunit, *B* gene encodes β-subunit and *P* gene encodes P14K (Fig. 2). The recombinant expression vector pET-24a(+) harboring the *ABP* genes (pET-*ABP*) was constructed and transformed into *E. coli* for NHase expression. The calculated molecular mass of recombinant NHase was in accordance with that of the bands by SDS-PAGE analysis (Fig. 3a lane 3). The NHase was successfully purified (Fig. 3a lane 4) and the specific activity was 438.5 U/mg. The molecular mass of NHase was nearly 106 kDa detected by gel filtration chromatography, indicating that the NHase is a tetramer (Fig. 3b).

According to our previous work [3, 5, 7, 8, 19–21] and others’ reports [4, 13, 22] about the NHase activators, the P14K is necessary for the functional expression of NHase in *B. pallidus* RAPc8, *C. testosteroni* 5-MGAM-4D and *P. putida* NRRL-18668. Since the amino acid sequence of NHase in *B. petrii* possessed a high similarity (more than 90 %) with that in *P. putida* NRRL-18668, it is proposed that the P14K in *B. petrii* is also necessary for NHase activation. Here, the NHase expressed by pET-*ABP* plasmid exhibited enough activity (more than 10 U/mg), indicating the successful expression of NHase and P14K, though the P14K band was not detected in SDS-PAGE (Fig. 3a lane 3).

### Successful expression of the activator P14K

The N-terminal residues of a protein are linked to its stability, which is conserved in bacteria [23, 24]. N-terminal lysine endows a protein short half-life, whereas glycine endows the protein long half-life (N-end rule) [25]. P14K, hardly detectable in SDS-PAGE, was successfully expressed in the NHase of *P. putida* through molecular modification in the light of N-end rule [21]. To investigate this phenomenon in NHase activator P14K from *B. petrii*, a his-P14K was designed, in which a his tag was attached to the N-terminus of P14K. Since the N-terminal amino acids of P14K (KDE sequence) were changed to the GSS sequence of the designed his-P14K, the his-P14K would be more stable in terms of the N-end rule. The plasmid pET-*AB(his-P)* including NHase encoding genes *A* (α-subunit), *B* (β-subunit) and *his-P* (his-tagged P14K) was transformed into *E. coli* and was applied for NHase expression (Fig. 2). As a result, the his-P14K was successfully expressed (detectable in SDS-PAGE) (Fig. 3a lane 5) and the enzymatic activity detected in the cell-free extracts was at the same level with that of the original NHase (120.5 U/mg). Although NHase was successfully purified (Fig. 3a lane 6) and the specific activity was 430.1 U/mg, the amount of P14K expression was still low (Fig. 3a lane 5).

To investigate the influence of the his tag on the C-terminal of P14K, a P14K-his, adding a his tag at the C-terminal of P14K, was designed. The transformed cells carrying the pET-*AB(P-his)*, a plasmid including NHase encoding genes *A* (α-subunit), *B* (β-subunit) and *P-his* (C-terminal his-tagged P14K), were applied for NHase expression. As a result, the enzymatic activity detected was comparable to that of the original NHase, however, P14K-his was barely detected in SDS-PAGE (Fig. 3a lane 7). These results indicated that the C-terminal of P14K does not play a key role in the expression of P14K.

### High expression of P14K attached with a gst tag

**Fig. 4:**
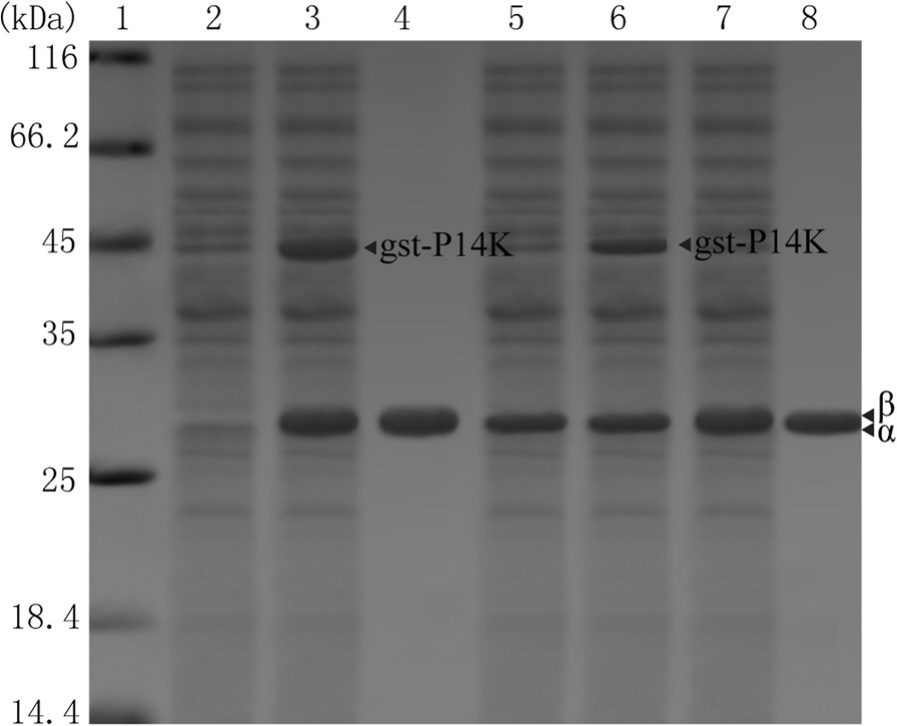
Stability analysis of P14K through SDS-PAGE. 1, mark; 2, control; 3, cell extracts (pET-*AB(gst-P)*); 4, purified NHase (pET-*AB(gst-P)*); 5, cell extracts (pET-*AB(his-P)*) stored for 2 days; 6, cell extracts (pET-*AB(gst-P)*) stored for 6 days; 7, cell extracts (pET-(A*)BP); 8, purified NHase (pET-(A*)BP)

The gst tag (26 kDa) encodes a highly soluble protein, which can help other proteins rapidly fold into a stable protein [26]. To produce a large amount of P14K, a gst-P14K, adding a gst tag at the N-terminus of P14K, was designed. The transformed cells carrying pET-*AB(gst-P)*, a plasmid including NHase encoding genes *A* (α-subunit), *B* (β-subunit) and *gst-P* (gst-tagged P14K), were applied for NHase expression. As shown in Fig. 4 lane 3, a large amount of gst-P14K was detected in SDS-PAGE. The enzymatic activity detected in cell-free extracts was at the same level with that of the original NHase. The recombinant NHase was then successfully purified (Fig. 4 lane 4), and the specific activity was 425.2 U/mg. Together with our previous work that the flexibility of P14K from *P. putida* plays an important role in NHase maturation [3] and the analysis that the molecular weight mass of gst (26 kDa) is nearly two times larger than that of P14K (14 kDa), it is suggested that the P14K possesses a high flexibility indeed.

Moreover, the protein stability of his-P14K and gst-P14K in the cell-free extracts was compared during storage at room temperature by SDS-PAGE analysis. As shown in Fig. 4 lane 5 and lane 6, the his-P14K band nearly disappeared after 2 days whereas the gst-P14K band decayed to 60 % after 6 days. The gst-P14K was far more stable than his-P14K, indicating that the thermal stability might be an important factor in the P14K expression.

**Fig. 5:**
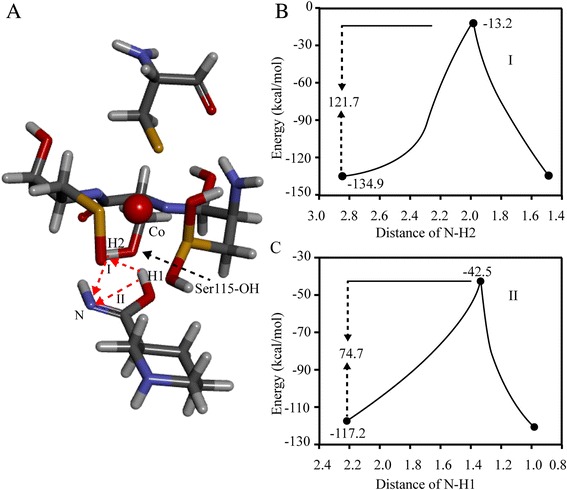
Energetic calculation of the proposed proton transfer pathway. **a** The structure of the proposed NHase catalytic model in the active center. The big red ball represents the cobalt ion. The Ser115 was shown by black dashed arrow. The H1 represents the proton of substrate transition state. The H2 represents the proton of Ser115(OH). The type I is the proton transfer pathway: H1(substrate) → H2(Ser115 of NHase) → N(substrate). The type II (direct transfer) is the proton transfer pathway: H1(substrate) → N(substrate). **b** Energy calculation of proposed transfer pathway (type I): the proton transfer pathway via the proton of OH(Ser115) (red dashed line). **c** Energy calculation of proposed transfer pathway (type II): the proton direct transfer pathway (not via the proton of OH(Ser115)) (red dashed line)

A characteristic motif CXLC-Ser-C has been discovered in all the α-subunits of NHases up to now [1, 14, 15]. The Ser between the two Cys in this motif is proposed to be involved in the NHase maturation [16–18], by which the proton is transferred to -NH_2_ (Fig. 1) [17]. These speculations indicated that the Ser was necessary for the NHase maturation. To investigate the necessity of the Ser, the Ser115 in NHase from *B. petrii* was researched. An energetic difference of the proton transfer via Ser115 (Fig. 5a type I) and the protein transfer not via Ser115 (direct transfer) (Fig. 5a type II) was compared through quantum chemical calculations using TRITON. As shown in Fig. 5b and c, the energy of the proton transferred to -NH_2_ via Ser115 (type I) (−13.2 kcal/mol) was more than that of the direct transfer (type II) (−42.5 kcal/mol), which indicated the type II pathway without the involvement of the Ser115 seemed easier to take place. Thus, a mutant NHase with the Ser115 mutated to Ala115 was designed to investigate the necessity of the Ser115, considering the structural similarity of the serine and alanine except for the -OH. The transformed cells carrying pET-(A*)BP, a plasmid including NHase encoding genes A* (mutant α-subunit with Ser115 to Ala115), *B* (β-subunit) and *P* (P14K), were applied for the mutant NHase expression (Fig. 4 lane 7). The enzymatic activity (45.3 U/mg) detected in the cell-free extracts was nearly 40 % of that of original NHase. The NHase was successfully purified (Fig. 4 lane 8), and the specific activity was 170.6 U/mg, also nearly 40 % of that of the purified original NHase. These findings suggested that the Ser in the characteristic motif CXLC-Ser-C of the active center of NHase is not necessary for the catalysis of NHase, however, it could kinetically facilitate the catalysis.

## Conclusions

The activator of NHase from *B. petrii* DSM 12804 was successfully over-expressed and the Ser115 was verified to be unnecessary for the catalysis of NHase by energetic calculation and site-specific mutagenesis. The strategy for the over-expression of P14K adopted in the paper might be beneficial for other proteins with difficulty in expression.

## Methods

### Bacterial strain and plasmids

NHase genes (*ABP*) were isolated from *B. petrii* DSM 12804 (accession number for genome, AM902716.1; α-subunit, CAP41751.1; β-subunit, CAP41750.1; P14K, CAP41749.1). The plasmid pET-24a(+) carrying the respective genes *ABP*, *AB(his-P)*, *AB(P-his)*, *AB(gst-P)* and (A*)BP was transformed into *E. coli* BL21 (DE3) cells (host strain) for heterologous expression.

### Plasmids construction

The NHase genes *ABP* of *B. petrii* were cloned through the polymerase chain reaction (PCR) with the primers A-up (NdeI recognition sites) and P-down (EcoRI recognition sites) (Table 1). The PCR reaction was conducted using the DNA polymerase KOD-Plus (TOYOBO) under the condition: a pre-denaturation at 94 °C for 5 min, 30 cycles of denaturation at 98 °C for 10 s, annealing at 48 °C for 30 s and extension at 72 °C for 90 s, and final extension at 72 °C for 10 min. The PCR product was analyzed from 1.5 % (w/v) agarose gel [27]. Afterwards, the cloned NHase genes *ABP* and the vector pET-24a(+) were treated by the NdeI and EcoRI, and the resultant *ABP* was then inserted into the pET-24a(+) for the pET-*ABP* construction. The pET-*AB(P-his)* construction was similar to pET-*ABP* with the primer pairs A-up and P-down(his) (EcoRI recognition sites). An overlap extension PCR was conducted to produce plasmid pET-*AB(his-P)* through two rounds of PCR. The first round of PCR was performed using the primer pairs A-up and B-down(his) and P-up(his) and P-down, respectively, the plasmid pET-*ABP* was used as the template. The second round of PCR was performed to produce the full-length *AB(his-P),* using the primers A-up and P-down and mixing equal molar amounts of the first-round products as template. The pET-*AB(gst-P)* construction was similar to pET-*AB(his-P)* with the primer pairs A-up and B-down(gst), and P-up(gst) and P-down (the g*st-P* gene synthesized by Sangon Biotech Ltd.). The plasmid pET-(A*)BP construction was conducted with the primers A*-up and A*-down using pET-*ABP* as the template, followed by DpnI digestion to degrade the template plasmid and then transformed into *E. coli* BL21 (DE3).

**Table 1:** Oligonucleotide primers used in this study

Name	Sequence
A-up	5‘-GGAATTC*CAT* *ATG*GGGCAATCACACACAC-3‘
P-down	5‘-CCG*GAATTC*TCAAGCCATTGCGGCAACGA-3‘
P-down(his)	5‘-CCG*GAATTC*TCAGTGATGATGATGATGATGAGCCATTGCGGCAACGA-3‘
B-down(his)	5‘-TGGCTGCTGCCCATATCTATATCTCCTTTCACGCTGGCTCCAGGTAGTCATC-3‘
P-up(his)	5‘-ATATGGGCAGCAGCCATCATCATCATCATCACAAAGACGAACGGTTTCCATT-3‘
B-down(gst)	5‘-CTATATCTCCTTTCACGCTGGCTCCAGGTAGTCATC-3‘
P-up(gst)	5‘-GAAAGGAGATATAGATATGTCCCCTATACTAGGTTA-3‘
A*-up	5‘-TCTTCGTCTGCACCCTGTGCGCGTGCTACCCATGGCC-3‘
A*-down	5‘-TGACGGCCATGGGTAGCACGCGCACAGGGTGCAGAC-3‘

The underlined code represents the gene start code or the Ser115 to Ala115 mutation. Italicized letters denote the NdeI and EcoRI recognition sites, respectively

### NHase expression, purification and activity assay

The recombinant *E. coli* cells carried the corresponding recombinant plasmids were cultivated in the TB medium to 0.8 of A600 with kanamycin (37 °C), and then 0.4 mM isopropyl β-D-thiogalactopyranoside (IPTG) was added. The transformed cells were then cultivated at 24 °C for 16 h.

The purifier AKTA instrument (GE Healthcare UK Ltd.) was used for proteins purification. All the purifications were kept in the potassium phosphate buffer (KPB, 10 mM, pH 7.5) at 4 °C. The cell extracts were centrifuged at 18000 × *g* for 10 min. Protein was preliminarily purified by the ammonium sulfate fractionation (30–70 %) followed by dialysis against KPB. The dialyzed protein ingredients of NHase were purified using a DEAE-Sephacel column (3 × 5 mL) (GE Healthcare UK Ltd.) equilibrated with KPB. The fraction containing target proteins was gradually eluted off by a linear concentration of KCl (0–0.5 M) during purification and then the collected proteins were pooled and followed by ammonium sulfate fractionation (70 %). After centrifugation, the dissolved precipitate (KPB containing 0.2 M KCl) was applied to a HiLoad 16/60 Superdex 200 prep grade column (GE Healthcare UK Ltd.) equilibrated with 0.2 M KCl-containing KPB. The enzymes were then eluted from the HiLoad 16/60 Superdex 200 prep grade column. The fractions containing the enzymes during the purification steps were revealed by SDS-PAGE.

The NHase activity was measured in a reaction system (500 μL) consisted of NHase (0.1 μg), the substrate (20 mM 3-cyanopyridine) and the buffer (10 mM KPB, pH 7.5), which was placed for 20 min at 20 °C and stopped by addition of 500 μL acetonitrile. The product concentration (nicotinamide) was detected with HPLC (high-pressure liquid chromatography) to measure the NHase activity [6]. The amount of protein that was capable of formation of 1 μmol nicotinamide in NHase catalysis was defined as one unit of activity.

### Homology modeling

The Modeller 9.7 [28] and the PROCHECK software (http://services.mbi.ucla.edu/SAVES/) were applied for the prediction of protein structure and stereochemical analysis. The final models that displayed good geometry (with less than 1 % of residues in the disallowed region) were used in this study.

### Transition state calculation

TRITON was used for modeling enzymatic reaction, which is freely supported by the National Center of Biomolecular Research (http://ncbr.chemi.muni.cz/triton/) consisting of three modules of MOPAC, MODELLER and DRIVER [29]. The optimal structure and the energy could be obtained and compared by activation energy calculation if the reaction pathway is reasonable. Notably, any improper structures were impossibly got when a proposed reaction pathway was difficult to perform [17].

## Abbreviations

*(A*)BP*: the NHase encoding genes including *A** (mutant α-subunit with Ser115 to Ala115), *B* (β-subunit) and *P* (P14K)
*ABP*: the full-length genes of NHase
Co-NHase: the cobalt type NHase
Fe-NHase: the iron type NHase
gst-P14K: a gst tag fused at the N-terminus of P14K
*his-P*: the gene of the protein his-P14K
his-P14K: a his tag fused at the N-terminus of P14K
NHase: nitrile hydratase
P14K-his: a his tag fused at the C-terminus of P14K
*P-his*: the gene of the protein P14K-his
